# The Transformation of Adolescent and Young Adult Oncological and Supportive Care in Canada: A Mixed Methods Study

**DOI:** 10.3390/curroncol29070406

**Published:** 2022-07-20

**Authors:** Jonathan Avery, Emily Wong, Christine Harris, Stacy Chapman, Serena Uppal, Shaayini Shanawaz, Annemarie Edwards, Laura Burnett, Tushar Vora, Abha A. Gupta

**Affiliations:** 1 School of Nursing, University of British Columbia, Vancouver, BC V6T 1Z4, Canada; 2 Department of Supportive Care, Princess Margaret Cancer Centre, University Health Network, Toronto, ON M5G 2C1, Canada; serenauppal2002@gmail.com (S.U.); sshanawaz@uwaterloo.ca (S.S.); tushar.vora@sickkids.ca (T.V.); 3 Canadian Cancer Society, Toronto, ON M4V 2Y7, Canada; emily.wong@cancer.ca (E.W.); christine.harris@cancer.ca (C.H.); annemarie.edwards@cancer.ca (A.E.); laura.burnett@cancer.ca (L.B.); 4 Department of Pediatric Haematology/Oncology, CancerCare Manitoba, Winnipeg, MB R3E 0V9, Canada; schapman@cancercare.mb.ca; 5 Department of Pediatrics and Child Health, University of Manitoba, Winnipeg, MB R3A 1S1, Canada; 6 Division of Medical Oncology, Princess Margaret Cancer Center, Toronto, ON M5G 2C1, Canada; abha.gupta@sickkids.ca

**Keywords:** adolescent and young adult, oncology, cancer, supportive care, mixed methods

## Abstract

Background: Due to ongoing disparity in the specialized care available to adolescents and young adults (AYAs) with cancer, this study aimed to understand the gaps and barriers to accessing care and preferences on types of solutions at a national Canadian level. Methods: A mixed-methods study involving an online survey and focus groups (FGs) was conducted among AYAs residing in different regions of Canada. Results: There were a total of 174 survey respondents, of whom the majority were between 30–39 years of age (*n* = 125, 71.8%). Of the 174 respondents, 36 (20.7%) participated in one of seven FGs. Triangulation of the results illustrated that AYAs are not appropriately informed about the long-term health risks of being treated for cancer and where/how to seek support. These results culminated into three themes: (1) the need for AYA relevant and timely information about health risks; by (a) producing health risk-related content with the AYA life stage in mind; (b) providing a guided “map” to help AYAs anticipate what they may experience, and (c) providing checklists to help AYAs navigate their experience; (2) need for tailored and timely supportive care including (a) establishing ongoing check-ins and (b) receiving navigation support, and (3) need for enhanced connections by creating (a) a space to gather, connect and seek mentorship and (b) a hub to access information. Conclusion: AYAs continue to lack sufficient support both during and following cancer and mechanisms are required to ensure longitudinal support is provided across jurisdictions and in all stages of the cancer journey.

## 1. Introduction

Approximately 7600 adolescent and young adults (AYAs) between 15–39 years of age are diagnosed with cancer in Canada annually and are in need of specialized AYA oncological and supportive care [1]. Fertility, socioeconomic worries (school/work-related, peer pressures, and financial well-being), and psychosocial (body image issues, sexual health) are concerns emphasized within the AYA population [1]. AYA cancers are generally treatable, resulting in an increasing denominator of survivors of AYA cancer that will need specialized longitudinal care to address these concerns [2]. Most AYAs receive oncological and supportive care at adult cancer centers. Pediatric models of survivorship care include life-long follow-up to screen for late effects of cancer treatment as well as offer longitudinal psychosocial support, including re-integration into society [3]. In comparison, adult cancer centers generally deliver care through disease-focused models in which high patient volumes and limited infrastructure challenge the ability to maintain long-term follow-up of survivors of AYA cancer [4].

A collaborative partnership between the Canadian Cancer Society (CCS) and the AYA Program at the Princess Margaret Cancer Centre (Toronto, ON, Canada) emerged to understand the concerns of AYA survivors and the type of support they require, independent of locus of care. Building on our earlier work [5] the main objective of our study was to explore the gaps and barriers to accessing oncological and supportive cancer care and preferences on future types of solutions at a national level.

## 2. Methods

A mixed-methods approach was used including an online survey and virtual focus groups (FGs). Ethics approval was obtained from the University Health Network (UHN) research ethics board (CAPCR #20-5080).

### 2.1. Study Participants and Setting

**Figure 1:**
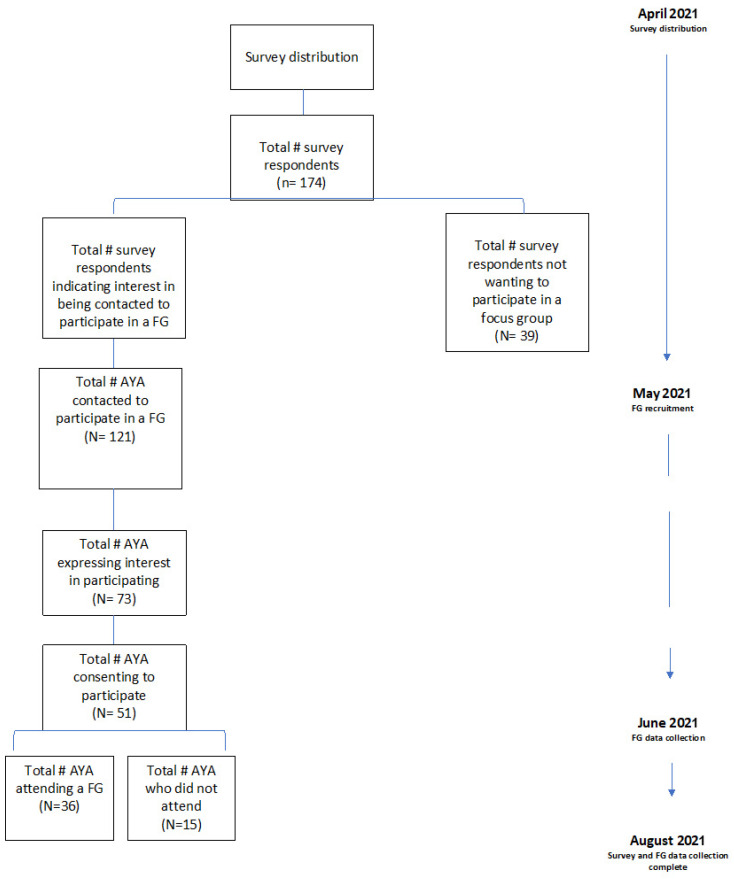
Participant recruitment flow chart.

Fluent English and French-speaking AYAs (age 19–39 years) residing in different regions of Canada about to start cancer treatment, receiving cancer treatment, in post-treatment follow-up care, or living with cancer as a chronic illness, (i.e., a diagnosis of advanced/metastatic disease) were recruited. Canada has two official languages (English and French) and therefore we sought to recruit individuals who spoke each official language. We also recognized that the needs and experiences of AYA could vary at each stage of the illness experience from treatment to post-treatment follow-up care and for those living with cancer as a chronic illness. To capture this range, this study was open to AYA at any stage of the journey. Canada is also geographically divided into 10 provinces and 3 territories within which comprehensiveness, design, and delivery of cancer control programs vary [6]. Variation in cancer control programs could impact the experiences and needs of AYA. To capture this possible variation, we divided Canada into five regions (Appendix A) to enable us to recruit a heterogeneous sample of AYA from different regions to ensure geographic diversity in our sample. Since the age of consent varies between 18 and 19 years of age depending on the province and/or territory, we chose to recruit AYAs between the ages of 19 and 39 to avoid any concerns with the age of consent. Data collection and recruitment for the survey and FGs occurred between April and August 2022 (Figure 1).

### 2.2. Survey—Recruitment, Execution, Analysis

AYAs were informed about the survey via email distribution lists and partnered networks via CCS. CCS is a national charity that unites and inspires all Canadians to take control of cancer and as such, has relationships with provincial and territorial cancer control programs across the country. Through these networks, as well as social media, (i.e., Twitter, Facebook, Instagram), the survey was disseminated to AYA participants. A convenience sample of approximately 150–200 AYAs was sought. A digital consent form was attached to the beginning of the online REDCap survey implying consent if the participant completes and submits the survey.

The survey included two sections: (1) demographics and (2) questions regarding AYA oncological and supportive care. The oncological and supportive care section sought to understand the informational, physical, and psychosocial concerns as it relates to the illness experience, gauge whether the care meets their needs, and collect ideas on future types of solutions/programs/tools to address unmet needs. The survey was first written in English and then translated by a professional transcriber into French. Survey data were analyzed by a member of the CCS Team (EW). Descriptive statistics (frequencies, percentages) were calculated using Excel pivot tables and inferential statistics were calculated using SPSS. Logistic regression was used to assess the variables associated with the availability of supportive care in community. Only fully completed surveys were used in the data analysis; respondents had the option to choose not to answer survey questions.

### 2.3. Focus Groups

On the final page of the survey, respondents were asked if they were interested in participating in an FG, and those who answered “yes” were asked to provide their names and contact information. We sought to recruit up to 80 AYAs to participate in an FG (up to 10 FG of 8 participants/FG) with each FG populated by AYA from the same region. To maximize representation across each region of Canada, potential participants were categorized by phone area code to help us populate focus groups by geographical region. We sought participant representation from every region. Potential participants were approached/contacted by phone by a member of the research team to discuss the FG portion of the study and to seek informed consent via REDCap. We continued to recruit for each FG until we had a maximum of 8–10 confirmed participants per FG. We aimed to over-recruit with the expectation that some individuals would consent but not attend. In cases when we were unable to secure 8–10 participants, we continued to recruit up until the day before the scheduled FG to maximize the opportunity to reach our recruitment targets.

FGs occurred virtually using the Microsoft Teams platform. Each FG was co-facilitated by the research lead (JA) and a member of the CCS team (CH). Other members from each team also partook in FGs as silent observers to take notes on what they observed. An FG guide was created to facilitate discussion (Table 1). The development of the guide was informed by the layout and questions asked on the survey. As a token of appreciation, participants received a $25 coffee gift card for their participation.

FGs were audio recorded and transcribed verbatim removing all participant identifiers from the audio recorded transcripts. Thereafter, a series of six procedural steps outlined by Braun and Clarke’s version of thematic analysis was followed [7]. FG transcripts were independently coded by four members of the research team (JA; SC; SU; SS). These four members met weekly to discuss their overall impression of the data and to develop an agreed-upon coding framework. Qualitative data analysis software NVivo 10TM version 12 was used to help organize codes, categories, and themes.

## 3. Results

There was a total of 174 survey respondents, of whom, most were between 30–39 years of age (*n* = 125). Of these, 36 (20.7%) participated in one of 7 FGs. More individuals identifying as women (*n* = 31, 86.1%) and between the 30–39 years of age (*n* = 26, 72.2%) participated in an FG (Table 2). Triangulation of the results from the survey and FGs illustrated that AYAs are not appropriately informed about the long-term health risks of being diagnosed and treated for cancer and lack the guidance to know where/how to seek support. Results are culminated under three themes: (1) need for AYA relevant and timely information about future health risks; (2) need for tailored and timely supportive care, and (3) need for enhanced connection; with recommendations emerging from the FGs articulated as sub-themes.

### 3.1. Theme 1: The Need for AYA Relevant and Timely Information about Future Health Risks

Of 174 survey respondents, 65% (*n* = 113) felt they did not receive an appropriate amount of information about the long-term health risks associated with their treatments, with only 13% (*n* = 22) reporting that they received health risk information at three different points—before, during and after treatment (Table 3). Fear of cancer recurrence (FCR), fear of a new and/or different cancer and concerns regarding emotional/mental health were ranked as the top three long-term health concerns by responders, at 89.7% (*n* = 156), 86.2% (*n* = 150), and 86.1% (*n* = 150), respectively, when combining scores of very concerned and somewhat concerned.

In the FGs, AYAs endorsed similar sentiments. Many participants articulated that they were not well informed about the longer-term future health risks prompting many fears including FCR and/or the onset of new/secondary cancers. A participant from FG 4 said: *“a lot of the focus was on acute side effects… but beyond that… chances of secondary cancers or possible heart conditions… weren’t… discussed in depth…”*. These sentiments led to several recommendations from FG participants, grouped under the following: (a) producing health risk-related content with the AYA life stage in mind; (b) providing a guided “map” to help AYAs anticipate what they may experience next; (c) providing checklists to help AYAs navigate their experience.

(a) Producing health risk-related content with the AYA life stage in mind

FG participants felt their stage of life was underrepresented in health educational material. Images and information contained in pamphlets represented older adults and statistics about long-term risks were associated with research conducted on older populations. Participants felt this information did not represent their stage of life and was therefore uninformative. Producing health risk-related content reflecting the AYA life stage was recommended. A participant from FG 1 said: *“You talked about the brochures and the fact that [you are] looking at older people, and you can’t relate to that… can there be some sort of resource… [to] target the information… [to] my demographics…”*.

(b) Having a guide to help AYAs anticipate what they may experience next

FG participants were overwhelmed by the amount of education about the consequences of cancer treatment to know when/how to review and apply this material. To understand how to apply the health risks information provided, participants suggested having some sort of guide through the journey so they could better anticipate the types of challenges that lay ahead. Having a *“… how to get started with cancer, some recommendations to help [young] people, because yes, if it’s your first time [with cancer], you have no idea what you’re doing…*” was a suggestion provided by a participant from FG 6. *“…having someone [or something] from the very beginning, from the moment you get diagnosed… to walk you through what to expect [as a young adult] with cancer”* was suggested from an FG 5 participant. It was this “lack of knowing” that prompted a participant from FG7 to recommend the need for *“…a higher-level map… to understand what the next five years could look like…”* to guide them through each step of the journey.

(c) Providing checklists to help AYAs navigate the experience

Our FG participants articulated that information on the long-term health risks, if provided, was typically offered just once either before or soon after starting treatment, leaving many participants uncertain about future health risks. Moreover, participants felt some HCPs were not apt at talking about cancer with AYAs or were not always transparent/forthright/or were too busy to discuss the long-term implications of being a young adult with cancer. Providing AYAs with pre-defined checklists was suggested as a tool to better inform AYAs about the long-term health risks and to use them as guides to improve communication with oncology teams. To have *“a survey [checklist] at the beginning of my treatment… maybe revisited…with whoever [the specialist] you’re seeing at that time would be great…”* (FG 7) to become better informed and to develop a better rapport with their oncologist. To have *“… some sort of checklist of what you might need, like a click box and maybe there’s a catalog or something. And then you click on what area you might want more information on…”* was suggested by a participant from FG 3. Participants expressed that some treatment centers already have these types of checklists but are unfortunately not well utilized.

### 3.2. Theme 2: The Need for Tailored and Timely Supportive Care

Of the survey respondents, 75% (131/174) reported that they required some type of supportive cancer care, yet 67% (*n* = 117) felt they did not receive any or enough information about supportive care services/programs/resources irrespective of where they were in their cancer journey: at the time of diagnosis, during and/or after treatment (Table 2). More than half of respondents (56%, *n* = 97) said they did not know if the supportive care they would like to access was available in their community. In addition, between one-third to one-half of respondents (32–50%) were not interested in accessing the services that were available. 

Our FG results provided similar assertions. The needs of AYAs are in constant flux and the provision of supportive care needs to keep up with the rate of change. Yet, supportive care information was typically offered just once either before or soon after starting treatment, even though many endorsed needing that support throughout their journey. Some participants felt their needs were also dismissed and had to “chase” for support and/or advocate for their needs. A participant from FG2 said: *“I did end up having to chase for counselling after my treatment ended… And so, I asked twice to see someone and then finally they referred me”*. These experiences led to several recommendations from our FG participants, grouped under the following sub-themes: (a) the need for ongoing check-ins to keep up with shifting needs; (b) receiving navigation support.

(a) The need for ongoing check-ins to keep up with shifting needs

The need for continuous prompting or check-ins was a common recommendation among participants to ensure information and support was available to keep up with the shifting needs of AYAs. *“a couple of people on rotation to do like a bi-weekly check in or a monthly check-in…”* to *“… prompt you on things that you may or may not be thinking about…*” (FG3); *“… to be contacted by the cancer agency once the dust has settled and you’re ready to talk…”* (FG5). As this participant illuminates, not every individual was ready to talk about their concerns or to access support when it was offered to them. On-going check-ins were a recommendation to help AYAs link up to resources to keep up with their shifting needs.

(b) Receiving navigation support

Participants recommended having access to a diverse array of “AYA” navigators with different specialties so they would not have to chase after support. Access to a wide variety of navigators would also help address any barriers to access such as any stigmatization felt that AYAs should be capable of returning to daily life with ease after treatment—sentiments expressed by a participant from FG 2 who felt they were not allowed to bring up their post-treatment concerns. *“… a key advantage to having [navigators] within our healthcare system… it will give us permission to talk about what we think we need…[and] they will connect you with the right person…”*.

### 3.3. Theme 3: Need for Enhanced Connections

From the survey, respondents were asked their preferred option for receiving information and supportive care. An open-ended question followed asking them to describe/elaborate on their chosen answer. Over half (58%, *n* = 101) preferred a hybrid model of online/virtual and in-person care with 35% (*n* = 61) wanting in-person-only interactions. Only 7% (*n* = 12) preferred virtual care alone. Respondents living in rural areas (OR 4.2, CI 95%: 1.64–10.75; *p* = 0.003) and small towns (OR 3.6, CI 95%: 1.50–8.42; *p* = 0.004), were more likely than those living in metropolitan areas to report that the type of supportive care they would like is not available in their community.

When explored in more depth during FGs, participants were adamant that developing a hybrid of virtual/online and in-person means of providing information and supportive care would be ideal to enhance access regardless of geographical location. These results led to several recommendations from our FGs, grouped under the following sub-themes: (a) a space to gather, connect, and seek mentorship; (b) a hub to access information.

(a) A space to gather, connect, and seek mentorship

Participants suggested creating a dedicated physical or virtual space where AYAs could gather, connect, and mentor one another. Connecting with other AYAs with lived experience going through the challenges of being diagnosed and treated for cancer at any stage of the trajectory was highlighted. Some participants described this space as an online virtual hub or as an *“…app… where [young] people who have or are going through it [cancer] can talk to you about it…”* (FG2). Other participants described this type of connection as a physical place that offered *“… a big brother, big sister kind of [program] at the hospital…”* to connect and gain guidance from other AYAs with cancer.

(b) A hub to access information

Participants were candid that they wanted a central hub to search for credible information and where/how to access resources. A space described as a *“…website with a search engine or in an app, where I can just type in my question and get it answered or find a video…”* was suggested by a participant from FG 6. A participant from FG 1 suggested having a place *“… where you can get the right information when you need it most…”* as a key feature of this space. This notion of retrieving credible information and searching for where/how to access resources at “click” of a button was described as an AYA preference given the tech savvy nature of this generation.

## 4. Discussion

Our study puts forth several recommendations to better prepare AYAs for living with and/or beyond cancer. Previous research has corroborated that AYAs with cancer report not feeling prepared for the eventual reality of living with and/or beyond cancer [8,9] requiring a universal “AYA” approach to inform and educate this population. Our findings provide a similar recommendation, however, expand by providing reflections on a national level as well as offering tangible solutions.

AYAs are offered plenty, and occasionally, too much or not enough of the right type of support at diagnosis. However, our study has confirmed that ongoing “check-ins” during cancer therapy are needed once the “dust settles”, and that these prompts need to come from the healthcare teams to mirror the progression of the cancer journey alongside the parallel changes in the AYA life. For example, the reality of the diagnosis may have solidified only after treatment has started such that decisions regarding school, work, and relationships can be processed much better than during that initial disclosure visit of “you have cancer”. Opportunities for recurrent conversations require structured approaches from health care systems. Similar efforts are required in survivorship wherein overwhelming concern surrounding the fear of cancer recurrence as expressed in this study mandates the need for systematic support in survivorship across all diagnoses. Possibly, navigators trained in the fear of cancer recurrence [10], mental health issues in survivorship including post-traumatic stress disorder [11], as well as occupational therapists with expertise on return to work [12] need to be employed at a national level. One of the main issues is that late effects of survivors of young adult cancers are not routinely collected [13], which places an even stronger need for open discussion about the long-term risks from trained professionals and/or navigators. Organizations such as the American Society of Clinical Oncology and the National Comprehensive Cancer Network (NCCN) have established professional guidelines to address issues related to the late-treatments effects for AYA diagnosed with cancer. These guidelines recommend taking a risk-reduction approach that invites two-way communication and facilitates referral to age-appropriate resources. So, even if the rate of late effects is uncertain, an open two-way dialogue is recommended, especially if the issue/concern is relevant/meaningful to the AYA patient (see Perez et al., 2020) [14]. Follow-up for AYA should be personalized and tailored according to the personal story of their path of care. Peer-driven support could also facilitate this process. Peer coaching and navigation also emerged as a key solution where AYAs could be trained to guide one another through the difficulties faced. Some organizations do offer this type of peer-driven support but not all are specific to the AYA population [15].

Our participants were candid that tools developed should incorporate design features that consider the ways AYAs consume information. Check-ins, checklists, and prompts are tools that some regional cancer programs and cancer centers have adopted [16,17] but are not all designed with the AYA population in mind [18]. A number of national and international organizations have developed their online/virtual hubs for AYAs to gather, receive mentorship, and retrieve information and resources [19,20]. In addition, some regional cancer centers in Canada and across other jurisdictions have their own dedicated programs that provide space for AYAs to gather and connect [4]. However, participants of our FGs illustrated that many are not aware these programs exist or were not specialized enough to meet their needs.

Social isolation again was an important problem highlighted by our participants [21], mandating improved mechanisms for connecting AYAs. A hybrid model of both in-person and virtual supports is preferred, necessitating national, regional and local investment. A co-design process with AYAs should be pursued to identify and develop strategies and interventions to address the challenges discussed in this paper and facilitate access to AYA tailored supports.

This study has some notable limitations. Among the 174 survey respondents, under 25% participated in an FG. Although we reached our recruitment targets for our survey, we were unable to reach our intended sample size for the FGs because of participant attrition. Even so, we are comfortable with our FG results, and we did not feel additional recruitment would yield new insights unless we purposely recruited a more diverse array of AYA. Respondents of both the survey and participants of the FGs do not reflect Canadian diversity regarding gender, and race, with notable no representation from the Yukon, Nunavut, and Northwest Territories. Indigenous peoples in Canada, the Black community and other minority groups continue to be underrepresented in cancer care research and this will need to be addressed in future research [22]. In addition, we did not explore the needs or experiences of those AYAs between 15–18 years of age. These younger AYAs may have experiences and needs that were not observed in our study; however, they are commonly cared for by pediatric institutions where support systems may vary. However, we feel the themes that have emerged speak to the overall life stage of AYAs and their shifting needs. With that being said, the ways in which these strategies are implemented could vary depending on gender, and other criteria such as the age and life stage of the AYA, cultural background, and education level. Further research is being explored to specifically focus on underrepresented populations.

In summary, our results pinpoint several directions to advance the delivery of oncological care for the AYA population. Future research could explore the implementation and feasibility of the development of a guided approach, continuous prompting, and ways to inform and integrate the different digital and in-person spaces that AYAs can gather, connect, and mentor. Future research should also explore clinician perceptions to ensure that what is developed can be integrated into current clinical practice structures.

## Figures and Tables

**Table 1:** Focus group script: themes, questions, and probes.

Part 1—AYA Supportive Care	*Questions* Can you talk about the information and education you received about the long-term impact and health risks associated with your cancer treatments? Can you talk about the information you received about supportive care services/programs/resources? When did you receive information about supportive care services/programs/resources? *Probes* i. What sort of education would be helpful for you? During treatment or after treatment? ii. What would it look like for you? Health care professional? Via an application? Brochure? iii. Thinking about your own diagnosis and treatment, can you discuss the follow-up care you received after your cancer treatment? What kind of support were/are you hoping to receive? Did you receive it? In what form?
Part 2—Types of supportive care currently being accessed Preamble: In terms of delivery of guidelines, appointments, and education, we are going to discuss 3 (2?) different approaches to delivery of supportive and survivorship care information. Hard-copy information vs. online delivery of information.	What types of support are you currently accessing? These supports could include anything from services/programs/resources that help address any emotional/psychological difficulties you have experienced or are currently experiencing. These supports could also include spiritual counseling and even some of the more practical supports, (i.e., managing finances; education/career counseling, transportation, nutrition, exercise and physical fitness). It can also include information on symptom management of the side effects from cancer treatment, and sexual/reproductive health. *Probes:* i. *What support service did you use the most, or like the best? What did you like about it?* ii. *What did you dislike about these supports?* iii. *How did you access these programs/supports?* iv. *How would you improve these supports?* How can we improve your ability to access these programs/supports? *Probes:* (i) *Where should the programs/supports you need be available?* (ii) *How should you discover these programs/supports?* (iii) *How would you like to access them?* *Would providing these supports virtually help enhance access? What would this look like?*
Models of care Preamble: In terms of delivery of supportive care, we are going to discuss different approaches to delivery. This includes in person or virtual/online approaches.	If you could create the “ideal” service for young adults—which would help you access relevant cancer information and supports—what would that look like? *Probes:* (i) *Do you want in-person options? Are you looking more for retreats, or opportunities to build social connections? How would you have liked to or want to be connected to your peers?* (ii) *Would you like someone to guide you through the service? Or would you prefer to navigate it on your own?* (iii) *Would you want hard-copy information you can take home?* *How would you incorporate virtual approaches? Would you want a website? An app? Email? Social media?*
Final comments	Are there any final comments that anyone would like to add that will help us to understand the best approach to provide AYA supportive care?

**Table 2:** Demographics of study participants.

Characteristics		Total Patients Survey (n, %) *N* = 174	Focus Group (n, %) *N =* 36
**Age**	19–24	17 (9.77)	1 (2.78)
	25–29	27 (15.5)	8 (22.2)
	30–39	125 (71.8)	26 (72.2)
	Prefer not to answer	5 (2.87)	1 (2.78)
**Gender**	Woman	143 (82.2)	31 (86.1)
	Man	27 (15.5)	5 (13.9)
	Non-Binary	2 (1.15)	-
	Transgender	1 (0.57)	-
	Prefer not to answer	1 (0.57)	-
**Region**	Central Canada (Ontario and Quebec)	94 (54.0)	15 (41.7)
	Western Canada (British Columbia and Alberta)	51 (29.3)	13 (36.1)
	Prairie Provinces (Saskatchewan and Manitoba)	18 (10.3)	5 (13.9)
	Atlantic Provinces (Newfoundland and Labrador, Prince Edward Island, New Brunswick, Nova Scotia)	11 (6.3)	3 (8.30)
	Territories	0 (0.00)	0 (0.00)
**Population Centre**	Rural (less than 50,000)	30 (17.2)	5 (13.9)
	Small Town (between 50,000–250,000)	40 (23.0)	10 (27.8)
	Large City (250,000–1 million)	44 (25.3)	8 (22.2)
	Metropolitan Centre (over 1 million)	59 (33.9)	13 (36.1)
	Prefer not to answer	1 (0.57)	-
**Cancer Treatment Stage**	Yet to start treatment	2 (1.15)	
	Currently in Active Treatment	54 (31.0)	8 (22.2)
	Completed Treatment and in Long-term Follow-up	95 (54.6)	23 (63.9)
	Discharged from Cancer Centre	19 (10.9)	5 (13.9)
	Unsure	4 (2.30)	-
**Time of Diagnosis**	<1 year	45 (25.9)	8 (22.2)
	1–3 years ago	70 (40.2)	16 (44.4)
	4–6 years ago	27 (15.5)	5 (13.9)
	≥6 years ago	31 (17.8)	7 (19.4)
	Prefer not to answer	1 (0.56)	-

**Table 3:** Survey responses.

**Question**	**Selection**	**N (%)**
**When did you receive information about long-term health risks?**	Before starting treatment	116 (66.7)
	During treatment	72 (41.4)
	Once finished treatment	43 (24.7)
	Not yet received detailed information	32 (18.4)
	Prefer not to answer	2 (1.15)
**From the (health risk) information received, was it?**	Not enough	92 (52.9)
	Just enough	54 (31.0)
	Too much	4 (2.30)
	Yet to receive detailed information	21 (12.1)
	Prefer not to answer	3 (1.70)
**When did you receive information about supportive care?**	Before starting treatment	76 (43.7)
	During treatment	85 (48.9)
	Once finished treatment	54 (31.0)
	Not yet received detailed information	33 (19.0)
	Prefer not to answer	3 (1.70)
**From the (supportive care) information received, was it?**	Not enough	100 (57.5)
	Just enough	54 (31.0)
	Too much	0 (0.00)
	Yet to receive detailed information	17 (9.77)
	Prefer not to answer	3 (1.70)
**Level of concern about each of the following health issue at this time:**	**Brain fog**	
	Does not apply to me	6 (3.45)
	I am not sure	3 (1.72)
	Not concerned at all	17 (9.77)
	Not very concerned	34 (19.5)
	Somewhat concerned	69 (39.7)
	Very concerned	45 (25.9)
	**Fertility**	
	Does not apply to me	14 (8.05)
	I am not sure	7 (4.02)
	Not concerned at all	32 (18.4)
	Not very concerned	17 (9.77)
	Somewhat concerned	38 (21.8)
	Very concerned	66 (37.9)
	**Mental and emotional health**	
	Does not apply to me	1 (0.57)
	I am not sure	2 (1.15)
	Not concerned at all	6 (3.45)
	Not very concerned	15 (8.62)
	Somewhat concerned	54 (31.0)
	Very concerned	96 (55.1)
	**Physical health**	
	Does not apply to me	2 (1.15)
	I am not sure	1 (0.57)
	Not concerned at all	6 (3.45)
	Not very concerned	27 (15.5)
	Somewhat concerned	54 (31.0)
	Very concerned	84 (48.3)
	**Fear of new/different cancer**	
	Does not apply to me	2 (1.15)
	I am not sure	5 (2.87)
	Not concerned at all	6 (3.45)
	Not very concerned	11 (6.32)
	Somewhat concerned	41 (23.6)
	Very concerned	109 (62.6)
	**Fear of cancer recurrence**	
	Does not apply to me	6 (3.45)
	I am not sure	1 (0.57)
	Not concerned at all	3 (1.72)
	Not very concerned	8 (4.60)
	Somewhat concerned	36 (20.7)
	Very concerned	120(69.0)
	**Sexual health**	
	Does not apply to me	3 (1.72)
	I am not sure	1 (0.57)
	Not concerned at all	16 (9.20)
	Not very concerned	29 (16.7)
	Somewhat concerned	61 (35.1)
	Very concerned	64 (36.8)
	**Managing finances**	
	Does not apply to me	6 (3.45)
	I am not sure	27 (15.5)
	Not concerned at all	38 (21.8)
	Not very concerned	39 (22.4)
	Somewhat concerned	64 (36.8)
	Very concerned	6 (3.45)
	**Return to work/career/school**	
	Does not apply to me	16 (9.20)
	I am not sure	2 (1.15)
	Not concerned at all	26 (14.9)
	Not very concerned	27 (15.5)
	Somewhat concerned	41 (23.6)
	Very concerned	62 (35.6)
	**Making/maintaining social connections**	
	Does not apply to me	2 (1.15)
	I am not sure	3 (1.72)
	Not concerned at all	35 (20.1)
	Not very concerned	36 (20.7)
	Somewhat concerned	64 (36.8)
	Very concerned	34 (19.5)
	**Support for caregivers, (i.e., my parents/spouse/siblings)**	
	Does not apply to me	15 (8.62)
	I am not sure	4 (2.30)
	Not concerned at all	37 (21.3)
	Not very concerned	32 (18.4)
	Somewhat concerned	56 (32.1)
	Very concerned	30 (17.2)
	**Child-care support**	
	Does not apply to me	69 (39.7)
	I am not sure	1 (0.57)
	Not concerned at all	46 (26.4)
	Not very concerned	24 (13.8)
	Somewhat concerned	18 (10.3)
	Very concerned	16 (9.20)

**Figure A1:**
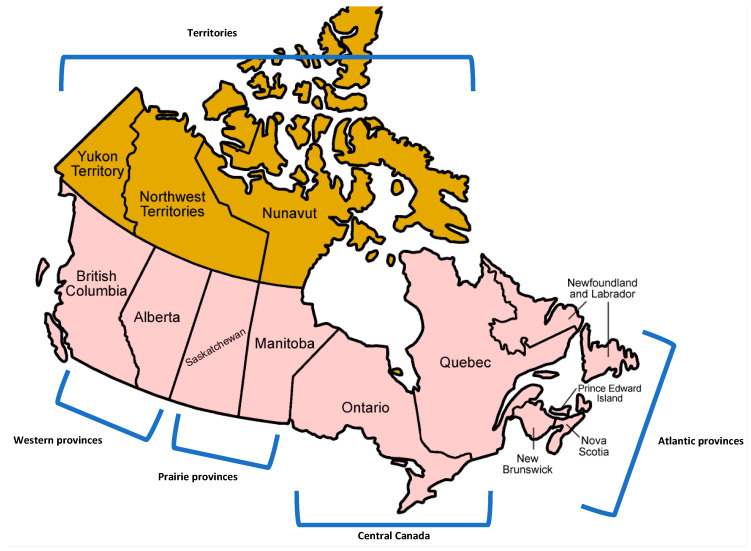
Regions of Canada.

## Data Availability

The data presented in this study are available on request from the corresponding author.
